# *‘It was nothing that you would think was anything’*: Qualitative analysis of appraisal and help seeking preceding brain cancer diagnosis

**DOI:** 10.1371/journal.pone.0213599

**Published:** 2019-03-22

**Authors:** Suzanne E. Scott, Clarissa Penfold, Smiji Saji, Sarah Curtis, Colin Watts, Willie Hamilton, Alexis J. Joannides, Fiona M. Walter

**Affiliations:** 1 Centre for Oral, Clinical & Translational Science, Faculty of Dentistry, Oral and Craniofacial Sciences, King’s College London, Guy’s Hospital, London, United Kingdom; 2 The Primary Care Unit, Department of Public Health & Primary Care, University of Cambridge, Cambridge, United Kingdom; 3 Clinical School, School of Clinical Medicine, University of Cambridge, Cambridge, United Kingdom; 4 Institute of Cancer and Genomic Sciences, University of Birmingham, Birmingham, United Kingdom; 5 Willie Hamilton, St Luke’s Campus, University of Exeter, Exeter, United Kingdom; 6 Department of Clinical Neurosciences, Division of Neurosurgery, University of Cambridge, Cambridge Biomedical Campus, Cambridge, United Kingdom; University of Sussex, UNITED KINGDOM

## Abstract

**Background:**

The patient’s interpretation of the events and decisions leading up to consultation with a healthcare professional for symptoms of brain cancer is under researched. The aim of this study was to document responses to noticing the changes preceding a diagnosis of brain cancer and living with them, focusing on appraisal of changes and the decision to seek (and re-seek) help, with attention to the psychological processes underpinning the appraisal and help-seeking intervals.

**Method:**

In this qualitative study set in Eastern and NW England, in-depth interviews with adult patients recently diagnosed with primary brain cancer and their family members were analysed thematically, using the Model of Pathways to Treatment as a conceptual framework.

**Results:**

39 adult patients were interviewed. Regarding the appraisal interval, cognitive heuristics were found to underpin explanations of changes/symptoms. The subtlety and normality of changes often suggested nothing serious was wrong. Common explanations included stress or being busy at work, or age and these did not seem to warrant a visit to a doctor. Explanations and the decision to seek help were made within the social context, with friends, family and work colleagues contributing to appraisal and help-seeking decisions.

Regarding the help-seeking interval, barriers to seeking help reflected components of Social Cognitive Theory, and included having other priorities, outcome expectations (e.g. ‘feeling silly’, not sure much can be done about it, not wanting to waste doctors’ time) and accessibility of a preferred healthcare professional.

**Conclusion:**

Application of psychological theory facilitated understanding of the influences on cognition and behaviour. The study highlights implications for theory, awareness campaigns and potential opportunities promoting more timely help-seeking.

## Introduction

The prognosis for primary brain cancer is poor. Only 40% of patients live for more than a year after diagnosis and less than 20% live for more than 5 years [1]. Furthermore, brain cancer results in the most life-years lost of any cancer [1,2]. In addition to poor prognosis, advanced disease may bring neurological disability from cancer-related operative brain injury and disease progression [3].

Brain cancer research has been neglected, with low levels of research funding compared to other cancers [4,5]; this includes research on how and when these cancers are diagnosed and the potential for more timely diagnosis and better patient experiences. In England, recent studies have shown that patients are often diagnosed after presenting as an emergency and/or after multiple visits to primary care [6]. Thirty-nine percent of brain/CNS cancer patients had three or more pre-referral consultations with a general practitioner (GP) compared with an average of 25% for all cancers [7]. However, little is known about the pathways to diagnosis from the patient’s perspective, including the events and decisions leading up to consultation with a healthcare professional (HCP).

In a Swedish study [8], researchers interviewed patients with malignant glioma and reported that patients may wait before consulting a HCP if their symptoms are ‘less-alien’ (i.e. common in everyday life), the patient experiences personality change or is prone to avoidance. However, that study was conducted post-treatment and focused on symptoms (headache, seizure, motor or sensorial dysfunction and mental dysfunction) that triggered help-seeking, rather than those that may have been experienced but not acted upon. A brief report from Scotland [9] indicated that interviews with patients tended to elicit histories of symptoms different to those recorded in hospital records, and called for more detailed study of patients’ and relatives’ experiences.

**Fig 1:**
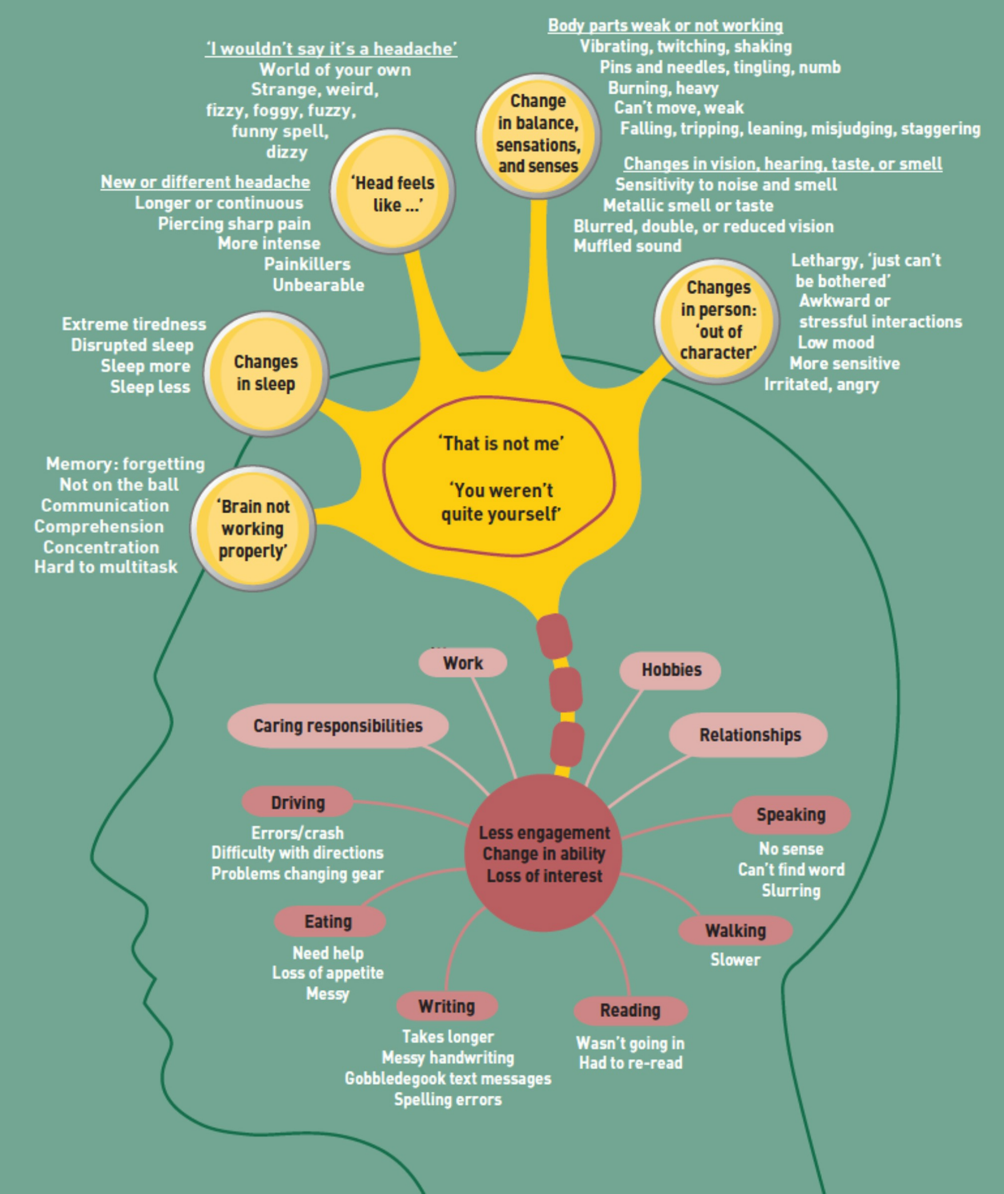
Multiple subtle changes preceding a brain cancer diagnosis.

In our new study, patients recently diagnosed with brain cancer and their relatives were interviewed to identify missed opportunities for more timely diagnosis [10]. The study found that people noticed multiple subtle changes preceding brain cancer diagnosis, including: changes in cognition such as speaking and writing, comprehension, memory, concentration and multi-tasking; changes in sleep; headaches and other head sensations that were not a headache; changes in senses, sensations and balance; and changes in personality or character. The changes could be somatic, but could also lead to less engagement, loss of interest, or a change in ability with daily living activities, relationships, work, hobbies, or caring responsibilities (see Fig 1). Patients described selective disclosure of these changes to their GP as they did not feel some changes were necessary for consultation, they forgot to mention the changes, were reluctant to give more details, or found the consultation too short to do so.

We do not know how people make sense of changes preceding a brain cancer diagnosis (especially for the whole range of changes beyond a headache and seizure), what underpins these appraisals, and why people decide to consult (or re-consult) a HCP prior to diagnosis of brain cancer. We need to further understand the processes of appraisal and help-seeking if we are to promote ways to enable a patient with subtle changes to consider seeking help and to convey the changes to a HCP.

**Fig 2:**
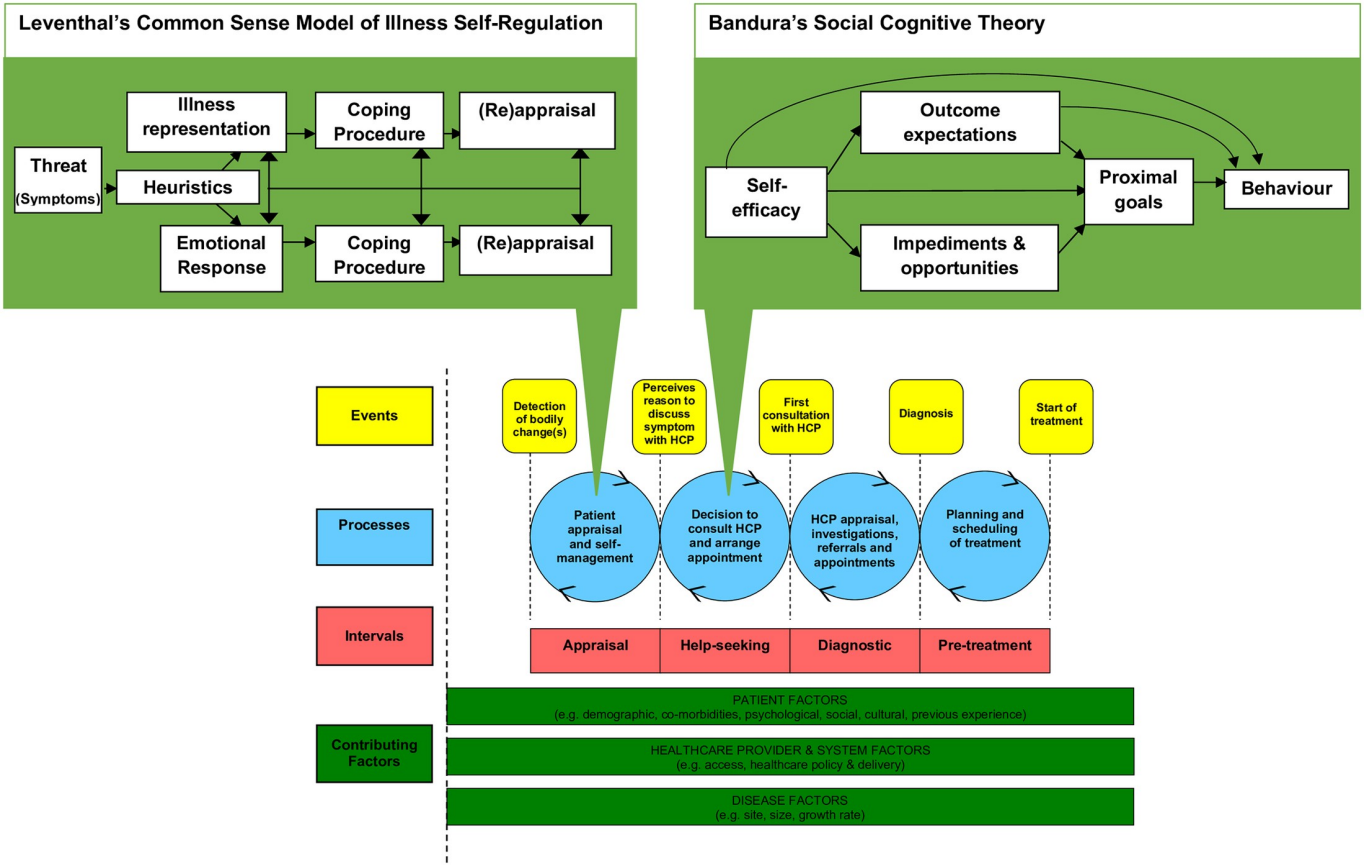
The integration of existing psychological theory into the Model of Pathways to Treatment.

We know from other cancers that appraisal of bodily changes and help-seeking decisions are important in the diagnostic pathway [11, 12]. The appraisal interval (the time from detection of a bodily change to perceiving a reason to discuss symptoms with a HCP) and help-seeking interval (time from perceiving a reason to discuss symptoms with a HCP to the first consultation with a HCP about their symptoms) together form the Time to Presentation (time from the detection of bodily changes to the first consultation) [12,13]. Time to Presentation is often the longest interval in the diagnostic pathway [14]. Existing psychological theories of health behaviour (e.g. the Common Sense Model of Illness Self-regulation (CSM) [15] and Social Cognitive Theory (SCT) [16,17] can be used to gain insight into the factors that influence the processes within the appraisal interval and help-seeking interval respectfully (see Fig 2) [13]. Briefly, the CSM notes that when bodily changes are unexpected or exceed a threshold of interference, a psychophysiological comparison occurs, comparing the current episode with memories of prior symptom episodes, other people’s past experience, and illness schemas. This, together with cognitive heuristics, (see Table 1) provides an explanation (representation) of the symptom [18]. The explanation and associated emotional response are used to plan ways of coping (IF-THEN plans), including whether a visit to a HCP is required [19].

**Table 1:** Cognitive heuristics that may underpin symptom appraisal.

Heuristic	Definition
**Age-illness rule**	*As individuals grow older*, *they increasingly attribute sensations to the ageing process rather than to illness*.
**Duration rule**	*Symptoms that are persistent or prolonged (compared to previous experience or expectations)*, *rather than short lived or intermittent*, *can indicate a level of seriousness*, *which in turn*, *can be a reason for urgently seeking help*.
**Novelty rule**	*Symptoms that are new*, *different*, *or incongruent (unexpected) with underlying schema rather than familiar*, *common*, *or similar to a co-existing chronic illness can be a key motivator to seek help*
**Optimistic Bias rule**	*Individuals have a generally optimistic bias in their interpretations (in keeping with previous experience) and will tend to make innocuous explanations rather than those that are life threatening*.
**Pattern rule**	*Compared to symptoms that are striking*, *severe or extreme*, *symptoms that are diffuse*, *mild*, *ambiguous or vague are less likely to be interpreted as indicators of illness or in need of prompt medical care*.
**Prevalence rule**	*Symptoms perceived to be prevalent in the community are more likely to be considered less threatening (i*.*e*., *minor rather than serious)*. *Conversely*, *symptoms that are seen to be rare are more likely to invoke concern and act as a motivator to seek help*.
**Rate of change rule**	*Compared to symptoms that are getting better/improving*, *stable*, *or decreasing in number*, *symptoms that are worsening*, *or increasing in number*, *and symptoms that have a sudden rather than gradual onset*, *can indicate illness and provide motivation to seek help promptly*.
**Severity rule**	*Symptoms that disrupt functioning indicate the presence of illness and/or the need for care whereas those that allow normal functioning will reduce motivation to seek help*.
**Stress-illness rule**	*Ambiguous symptoms are often discounted during times of acute stress and are more likely to be attributed to stress rather than physical illness*.

When a person perceives a reason to discuss a symptom with a HCP, whether or not they decide to actually seek help (and the time it takes them to do so), may be explained by anticipatory factors outlined in SCT. SCT suggests that behaviour is determined by self-efficacy (i.e. perceived ability to discuss the symptoms and get help), socio-structural impediments (e.g. personal barriers or aspects of the health care system), outcome expectations (perceived consequences of action) and current competing priorities (proximal goals). The aim of the current study is to document the reported responses to noticing and living with the changes preceding a diagnosis of brain cancer, focusing on appraisal of changes and the decision to seek, not seek and/or re-seek help, with attention to these psychological processes underpinning the appraisal and help-seeking intervals. This paper reports on data collected during the previous study [10].

## Materials and methods

### Design

In-depth interviews were conducted face-to-face with adults very recently diagnosed with primary malignant brain cancer (cerebral glioma), together with their relatives. The study was undertaken and reported in line with the Consolidated criteria for reporting qualitative studies (COREQ) [20].

### Procedure

The study protocol was approved by Cambridge South NRES Committee, East of England (REF: 16/EE/0179) and is explained in detail elsewhere [10]. Adults aged 18 and over, newly diagnosed (within 4 weeks) with primary brain cancer at Cambridge University Hospitals NHS Foundation Trust and The Walton Centre, Liverpool, UK were eligible for inclusion. Potential participants were identified and recruited by the neuro-oncology nurse specialists. Purposive sampling strategies were used to recruit a range of participants by age, gender and location to ensure a broad range of views and experiences. Interviews were continued until no new themes were identified.

Following written informed consent, and before treatment (brain surgery, with the exception of one participant who declined surgery) and histological confirmation, semi-structured interviews were carried out in patient’s homes with patients; their relatives were also often present. An experienced female Research Associate with doctoral level training in qualitative research (CP) used an interview topic guide (see S1 File) focusing on when and how initial symptoms were noticed; the language used to describe symptoms and changes over time; the participant’s decision-making and triggers to help-seeking; and experiences of the diagnostic process from the patient perspective. Interviews lasted between 45 and 90 minutes, were audio-recorded, professionally transcribed verbatim, checked and anonymised. Following surgery, details of diagnosis and WHO grade were obtained from medical records.

### Analysis

Transcripts were imported to NVivo 11 software to support coding and data organisation. Inductive thematic analysis commenced soon after the beginning of data collection [21]. The Model of Pathways to Treatment [12–13] was used as a conceptual framework to underpin the analysis. A coding frame based on this model was developed with, and applied to, the first few transcripts, and refined iteratively, applying a constant comparative method [22]. This framework initially focused on data-driven patient, healthcare/system or disease factors that contributed to the appraisal and help-seeking intervals. The transcripts were coded by one researcher (CP) and moderated by a further two (FMW and SC). The coded text was then analysed using more a more deductive approach (by SES, moderated by FMW), purposely focusing (but not restricted to) the concepts of CSM for symptom appraisal and SCT for barriers to seeking help as suggested in the Model of Pathways to Treatment. As the first consultation often did not result in referral, we also included subsequent appraisal (of the same or new changes/symptoms) and decisions to re-consult in the analysis of the contributing factors to the appraisal and help-seeking intervals.

### Checking for credibility

Key themes and sub-themes were agreed and refined through a series of meetings involving the ‘core’ researchers (from a range of clinical and non-clinical backgrounds), the two consumer members of the research team, and consensus meetings with the wider study team including neuro-oncology experts. Reflexive field notes were made following the interviews. In addition, we shared early findings at a workshop supported by our funder The Brain Tumour Charity, including GPs from London (n = 10), and patients (n = 7) and carers / family members (n = 9) affected by brain cancer drawn from across England. Four mixed facilitated focus groups (2 facilitated by FMW, 2 facilitated by SES) were undertaken to hear patient, carer and GP responses to the findings and how these compared to their own experiences. The group sessions lasted up to one hour each, were audio-recorded, transcribed verbatim, checked and anonymised. The transcripts from the focus groups were analysed separately and independently (by SS) then comparisons were made with the findings of the in-depth interviews to search for concordant, discordant, or new data. Interviews (as would be expected) went into more detail, but overall the themes from the workshop mapped on to those from interviews (see S2 File).

## Results

### Sample characteristics

54 people were interested in taking part. Of these, 3 decided not to be interviewed. Interviews could not be scheduled prior to surgery for 8 people. 43 people were interviewed, four of whom were subsequently diagnosed with a non-glioma tumour or secondary tumour. Here we report the data from the remaining 39 people diagnosed with primary cerebral glioma. The mean age of the patients was 53 years, and 18 (46%) were female. The majority (n = 30, 77%) were recruited from Eastern England. The commonest diagnoses were glioblastomas (n = 22, 56%) and astrocytomas (n = 9, 23%), located in the frontal (n = 20, 51%) or temporal region (n = 10, 26%) and WHO-graded as high-grade (n = 29, 74%). With regard to initial Time to Presentation (as determined from the patient account), very few (n = 8, 21%) consulted a HCP in less than 1 month. Eleven people (28%) waited 7–12 months, and 10 people (26%) waited over 1 year before consulting a HCP (see Table 2 for individual breakdowns).

**Table 2:** Characteristics of patients interviewed in this study.

ID	Gender	Age Band (yrs)	Interviewed with	Initial time to presentation	Diagnostic interval	Route to diagnosis
(01)	M	51–60	Spouse, adult daughter	> 12 months	1–4 weeks	1x walk-in-centre. 1x GP. 1x GP home visit. (after calling 111) 1x nurse home visit. 1x GP visit (different GP). 1 A&E (sent by GP).
(02)	M	51–60	Spouse	7–12 months	1–4 weeks	1x GP. 1x referral for OP-scan. 1x A&E (prior to scan)
(03)	F	31–40	Mother	7–12 months	7–12 months	2x GP (different GPs). 1x Mental Health Professional. 1 x A&E.
(04)	M	61–70	Partner	>12 months	1–4 weeks	1x GP. 1x A&E (sent by GP)
(05)	F	41–50	Spouse	>12 months	>12 months	2x GP (different GPs). 1x Optician (sent by GP). 1x A&E
(06)	F	31–40	-	>12 months	1–6 months	> 3x GP*. 1x SP-OP. 1x OP-scan
(07)	F	31–40	-	>12 months	1–6 months	3x GP. 1x OP-scan and SP-OP
(08)	M	61–70	Spouse	1–6 months	1–4 weeks	1x GP. 1x A&E (sent by GP)
(09)	M	61–70	-	7–12 months	1–4 weeks	1x GP. 1x A&E.
(10)	F	61–70	-	7–12 months	1–4 weeks	1x GP. 1x A&E.
(11)	M	71–80	Spouse	1–6 months	1–4 weeks	1x GP. 1x A&E (sent by GP)
(12)	F	51–60	Partner	>12 months	1–6 months	1x GP. 1x A&E (sent by GP). 1x Optician. 1x GP (sent by Optician). 1x private scan. 1x private SP-OP.** 1x GP. 1x SP-OP. 1x OP-scan. 1x SP-OP. 1x second opinion.***
(13)	F	31–40	-	>12 months	1–4 weeks	1x A&E
(14)	M	31–40	-	7–12 months	1–4 weeks	1x A&E. 1x GP.1x A&E.
(15)	M	51–60	-	7–12 months	1–6 months	1x A&E. 1x GP. 1x OP-scan. 1x Inpatient
(16)	M	61–70	Adult son	1–6 months	1–4 weeks	1x A&E
(17)	M	61–70	Partner	>12 months	1–6 months	1x A&E. 1x GP. 1x A&E.
(18)	F	61–70	Spouse	1–6 months	1–4 weeks	1x A&E
(19)	M	61–70	Spouse, adult daughter	7–12 months	1–6 months	1x GP. 1x A&E (after calling 111).
(20)	M	71–80	Spouse	7–12 months	1–4 weeks	1x A&E
(21)	M	51–60	Spouse	1–4 weeks	1–4 weeks	2x GP. 1x OP-scan.
(22)	M	61–70	Spouse	<7 days	1–6 months	1x A&E. 1x GP. 1x SP-OP. 1x A&E.
(23)	F	21–30	-	1–4 weeks	7–12 months	3x GP (different GPs). 2x SP-OP
(24)	F	41–50	-	7–12 months	7–12 months	1x GP. 1x walk-in centre. 2x GP (different GPs). 1x private SP-OP.
(25)	F	21–30	-	<7 days	7–12 months	1x emergency call (no A&E attendance). 1x A&E.
(26)	F	41–50	Friend	1–6 months	7–12 months	3x GP (2 different GPs). 1x private OP-scan.
(27)	F	71–80	-	7–12 months	1–4 weeks	1x GP. 1x A&E (after calling 111).
(28)	M	51–60	Partner	<7 days	1–4 weeks	1x A&E
(29)	F	41–50	-	1–6 months	1–6 months	2x GP. 1x OP-scan and SP-OP
(30)	M	71–80	Spouse	1–6 months	1–4 weeks	2x GP. 1x A&E (sent by GP)
(31)	M	41–50	Mother, Sister	>12 months	>12 months	>12x GP (at least 2 different GPs). 1x Optician. 1x A&E.
(32)	M	71–80	-	1–6 months	1–6 months	1x Optician. 1x private SP-OP. 1x GP. 1x OP-scan
(33)	M	51–60	Mother, Sister	1–6 months	<7 days	2x GP (different GPs). 1x OP-scan
(34)	M	41–50		1–6 months	1–6 months	2x GP (different GPs). 1x SP-OP
(35)	M	41–50	-	<7 days	1–6 months	2x A&E. 1x SP-OP.
(36)	F	21–30	Mother, another person	1–4 weeks	1–6 months	2x GP (different GPs); 1x OP-scan
(37)	F	31–40	-	<7 days	1–6 months	5x GP (different GPs). 1x ACU (sent by GP). 1x SP-OP.
(38)	F	51–60	Spouse, adult daughter	7–12 months	1–6 months	1x walk-in centre. 1x A&E (sent by walk-in-centre). 1x SP-OP. 1x A&E.
(39)	F	21–30	-	>12 months	1–6 months	1x A&E. 1x GP. 1x A&E (sent by GP). 1x SP-OP.

Key: SP-OP: specialist outpatient clinic; OP-scan: outpatient scan; ACU: Ambulatory Care Unit

* Patient was seeing GP “at least once a month” for other co-morbidities and is unsure how many time brain cancer related symptoms were mentioned.

** Unconfirmed diagnosis which was then dismissed

*** Definitive diagnosis

### The appraisal interval: Why and when people perceived a reason to discuss changes/symptoms with a HCP

Regarding the appraisal interval, three main themes are presented. The first, *‘Subtlety and normality of changes*: *I didn’t think anything of it’* and second *‘Something was wrong’* focus on overarching inferences as to whether or not something was wrong and the factors underpinning these suppositions, in particular the use of cognitive heuristics of symptom appraisal. Within these themes, specific explanations are given which outline the different explanations which sat alongside these general deductions. The explanations are discussed with reference to whether they were perceived to require healthcare attention, reflecting the CSM’s IF-THEN plans. Participants were often experiencing more than one change/symptom. Frequently, these were seen as unrelated issues (prior to diagnosis) and explanations would differ for different changes/symptoms, resulting multiple concurrent explanations. This could prompt the decision to discuss some symptoms with a HCP, but not others, resulting in selective disclosure to the GP. The third theme addresses the social context of symptom appraisal and the role of others in decisions to seek healthcare which applied across the interval. Quotes are displayed followed by details of the patient. If the quote is from a family member this is indicated before the quote, but the detail following the quote still refers to the patient.

#### Subtlety and normality of changes: *‘I didn’t think anything of it’*

Patients and their relatives spoke of how some changes were so subtle or slight that they seemed normal, often not even considered as a ‘symptom’ and definitely not something indicative of a serious problem such as cancer or requiring healthcare. In turn, they often gave very little thought to changes, and were not concerned as the changes could be easily explained. These interpretations follow the ‘Pattern rule’–a cognitive heuristic of symptom appraisal that suggests diffuse, mild, ambiguous or vague symptoms are less likely to be interpreted as indicators of illness or in need of prompt medical care.

‘When I was drinking a cup of tea it felt like a little shake on one side but it didn’t really enter my head that it would be anything … you know sometimes you’re tired and your arm shakes a bit, it just didn’t sort of … any alarm bells.’

[10, F, 61-70yrs]

‘I was at the sink doing some washing up and I kept leaning like that into the sink, very slowly. It’s funny, you just don’t even think, “I should go to the doctors with this,” you just don’t, it just seems like, “I’ll get over that”.’

[09, M, 61-70yrs]

‘Well, the numbness on my face is very slight, so probably don’t take as much notice of it […] I wouldn’t really take too much notice of again because it’s so slight.’

[24, F, 41-50yrs]

SPOUSE: ‘*He’d grate the gears and I’d go*, *“Gears” you know*, *you don’t associate oh my god*, *you must have a tumour because you can’t change the gears (laughter)*.*’*

[08, M, 61-70yrs]

Participants also spoke of how in hindsight they could see that the changes were linked to their subsequent diagnosis but because the changes/symptoms are relatively common, and not exclusive to brain cancer, there were numerous alterative and more likely explanations (reflecting the *‘Prevalence rule’*).

‘Even if I said the wrong word, we all say the wrong word or there’s a slip of the tongue anyway’.

[32, M, 71-80yrs]

‘You see all these symptoms, it can all be linked to it but they can all still be something else’.

[08, M, 61-70yrs]

SPOUSE: *‘I feel a bit guilty about that because it was obviously signs but I didn’t know they were signs*. *[…] We sort of found an explanation and a reason*. *[…] there was always a reason for how he was*, *so you didn’t question it because it was nothing that you would think was anything’*.

[01, M, 51-60yrs]

If changes only occurred infrequently and intermittently, meaning patients had phases without symptoms, they had less indication something was wrong or needed care (reflecting the ‘Duration rule’ see Tables 1 and 3). For changes that were mild and intermittent, patients could continue with their lives and there was little interference with work and other responsibilities, again acting as an indicator that there was nothing wrong (reflecting the ‘Severity rule’ see Tables 1 and 3) and the changes were not seen as a ‘threat’.

**Table 3:** Examples of cognitive heuristics in the appraisal of changes/symptoms.

Heuristic	Example	
	Contributing to nothing is wrong / nothing to worry about / didn’t consider seeking help	Contributing to something is wrong / not right / motivator to seek help
**Duration rule**	*‘It was happening now and again; I couldn’t pick my feet up*, *and then after a little while it would be alright and they’d come back again you know*.*’ [19*, *M*, *61-70yrs]* *‘It literally came on*, *a couple of seconds*, *swallowed my breath and it had gone*. *So it was very momentary*. *It wasn’t substantial*, *like*, *type of thing*.*’ [35*, *M*, *41-50yrs]* *‘So when I was talking it was like I was slurring slightly*, *but it would only last for about not even a minute and it would go*.*’ [38*, *F*, *51-60yrs]* *‘Yes*, *just only*, *you know*, *every now and then*. *Not something that’s*, *you know*, *happening every day*, *kind of thing*.*’ [39*, *F*, *21-30yrs]*	*‘And then as it was becoming more and more frequent we then … well*, *you said about going to the doctors again*.*’ [33*, *M*, *51-60yrs]* SPOUSE: ‘*I just thought*, *“There’s something really wrong here*. *He’s not improved over the 2 weeks”*.*’ [01*, *M*, *51-60yrs]* *‘I couldn’t get rid of these headaches*, *I got these headaches all the time*.*’ [19*, *M*, *61-70yrs]*
**Severity rule**	*‘I didn’t mention it to anyone*, *it was so nothing-y*. *[…] I think the auditory ones are so relatively un-interfering with life*.*’ [07*, *F*, *31-40yrs]* *‘I was still able to maintain the usual routine*, *so I’d be getting up at sort of 6*:*00ish*, *which was fine; it wasn’t problematic*.*’ [02*, *M*, *51-60yrs]* PARTNER: *‘That’s what the problem is really*, *nobody really noticed it because he was fine*, *going to work*.*’ [17*, *M*, *61-70yrs]* *‘You just focus on the thing that’s ailing you*, *whereas a bit of numbness here and there which isn’t always there*, *I suppose it’s not really stopping me doing anything*.*’ [24*, *F*, *41-50yrs]*	SPOUSE: ‘*He didn’t know what to do in the supermarket*, *basically*, *and came back with nothing and said*, *“I don’t know what happened but I couldn’t do that*. *I couldn’t do the shopping*.*”[…] And [DAUGHTER] was really worried about him*.*’ [02*, *M*, *51-60yrs]* *‘This brush kept dropping out my hand*, *so every time I tried to adjust it to go somewhere it dropped*. *And it must have been a dozen times*. *And then I thought*, *that was a turning point I suppose*.*’ [04*, *M*, *61-70yrs]* *‘It got to the stage where I couldn’t function*. *When I was at work*, *I just wanted to put my head on the desk*. *I felt I couldn’t drive*, *I just wanted to put my head down*, *so I thought*, *oh*, *I’m really going to have get seen*.*’ [24*, *F*, *41-50yrs]*
**Rate of change rule**	SPOUSE: *‘It’s been a slow thing*, *a bit like the episodes*. *It’s been a gradual change*, *hasn’t it*?*[…] I couldn’t say*, *“Oh*, *he’s suddenly changed in personality at this point*,*” it has definitely been a gradual change*.*’ [02*, *M*, *51-60yrs]* MOTHER: *‘A little while later he was perfectly fine*, *he was just talking*, *cos a friend of his phoned up and you were talking to her on the phone as if nothing had happened and I never really thought nothing about it*.*’ [31*, *M*, *41-50yrs]*	SPOUSE: *‘I mean there is no way you wouldn’t have been taken to hospital on that Friday*, *you know*, *because you were getting worse rather than better*.*’ [20*, *M*, *71-80yrs]* SISTER: *‘Within a week of mum and dad going on holiday and a week later*, *the deterioration in him was just huge*, *wasn’t it*? *[…] It was just like*, *well*, *he managed to string a sentence a week ago*, *you know*, *and then a week later*, *it was like*, *“My God*, *what’s happened to him*?*”‘ [33*, *M*, *51-60yrs]*
**Novelty rule**		*‘It just didn’t feel right and something I hadn’t ever experienced before*.*’ [22*, *M*, *61-70yrs]* *‘You don’t have that degree of memory loss and the loss of words*, *when you normally can articulate yourself in such a way that you’re understood*, *[…]*, *you know that’s not normal*.*’ [03*, *F*, *31-40yrs]*

Common explanations included stress or being busy at work, tiredness (reflecting the ‘Stress-illness rule’), or age (reflecting the ‘Age-illness rule’, see Table 1). Sometimes changes were explained by an event or circumstance that occurred shortly before they noticed the change. Thus, the chronology of the event and development of changes fitted a plausible timeline and offered an explanation. Reflecting the normal everyday explanations given to changes, a clear barrier to seeking help was the common belief that the changes people noticed simply did not warrant the attention of a HCP (see Table 4).

‘You wouldn’t go to the doctor’s thinking, well, you know, I’m grumpy, I keep rubbing my arm’.

[05, F, 41-50yrs].

‘I never, ever would have said to a GP I was more clumsy’.

[24, F, 41-50yrs]

**Table 4:** Specific explanations for changes/symptoms which could affect help-seeking.

**Common explanations unlikely to prompt help-seeking**	
**‘Just tiredness’**	*‘I put it down to a lack of sleep a lot of it*, *you know*. *I probably have been a couple of months more tearful’ [10*, *F*, *61-70yrs]* *‘Well I can’t sometimes find the words*, *the correct words*, *I’ll maybe talk round it a bit*, *and I’m sure that’s tiredness*.*’ [11*, *M*, *71-80yrs]* *‘I would misread words as well which I’ve never done that before*, *just little things like that*, *but you don’t think that’s significant*, *you just think you’re probably tired*.*’ [03*, *F*, *31-40yrs]* *‘We were doing an awful lot of overtime*, *so I just assumed I was tired*.*’ [36*, *F*, *21-30yrs]* *‘I thought that was my job because teachers are renowned for being tired […] Also*, *a lot of other people in my job will say*, *oh my God I’m so tired so I just thought*, *everybody is feeling like that*.*’ [13*, *F*, *31-40yrs]*
**‘Stress’ at home or at work**	*‘I was helping with my mum*, *which I wanted to do*, *…*, *but then we had a lot of trouble with my son and my daughter*, *and I then got this headache*, *so I just put it down to stress because I’d never felt stress like it’ [24*, *F*, *41-50yrs]* *‘If I concentrated on something*, *then I could quite easily get blurred vision but I didn’t take any notice of it because I just thought I was getting a bit stressed out at work and it was all to do with stress’ [08*, *M*, *61-70yrs]*. *‘I just put feeling tired down to the fact that it was a [long] engagement…*. *Yeah*, *weddings are stressful actually*, *they really are*. *They’re utterly joyful but stressful as well*.*’ [12*, *F*, *51-60yrs]*
**‘Age’**	*‘I did notice I was a little bit tired*, *but then I thought it was my age*. *[11*, *M*, *71-80yrs]* *‘I started napping in the afternoon*, *so I thought it was*, *“Oh*, *I’ve hit 50 now*.*” [38*, *F*, *51-60yrs]* *‘I was at work and I couldn’t type letters in the right order and I was getting frustrated*, *but*, *because we’re all in our 60s or 70s at work […] we just laughed about getting old sort of thing as you do*.*’ [10*, *F*, *61-70yrs]*
**Recent events**	*‘So*, *decorated two rooms*, *no problem*, *but getting headaches from painting*. *“Ah*, *it’s the bloody paint*, *forget it*.*”‘ [32*, *M*, *71-80yrs]* *‘About a couple of weeks before it started I got hit on the elbow with a mallet at work*. *[…] I thought that’s what was causing it*, *some sort of nerve damage or something like that*.*’ [33*, *M*, *51-60yrs]* FRIEND: *‘I think there was quite a few of us who noticed*, *but we put it down to the fact you were on these painkillers*.*’ [26*, *F*, *41-50yrs]*
**Less common explanations which only sometimes prompted help-seeking**	
**Existing medical problem**	*‘It came back that I was diabetic and then I put everything down to being diabetic*. *[…] It’s mostly down my left leg where I get pins and needles and in my left foot*, *[so] I thought it was bad circulation because I’ve got diabetes as well*.*’ [06*, *F*, *31-40yrs]* *I’ve got white finger*, *you know*, *where my fingers go numb and white*, *so I suppose I put it down to that*,*’ [08*, *M*, *61-70yrs]* *‘I thought*, *“It’s got to be this hernia*. *I’ll have to go down and see the doctors*,*” [Then] I thought*, *“Well*, *perhaps I need to just rest it all and be gentle with it*, *and everything else will improve around it*,*” but it didn’t*.*’ [33*, *M*, *51-60yrs]*
**New or existing Anxiety or Depression**	*‘I just can’t concentrate*, *I can’t concentrate whatsoever but I thought that was down to*, *again*, *the depression*.*’ [06*, *F*, *31-40yrs]* *‘I got myself in such a state that I thought I’m going to have to go back to the doctor’s and get these antidepressants again*.*’ [05*, *F*, *41-50yrs]* *‘I put it down to the fact that sort of like mental health problems*, *that’s why my memory wasn’t so good*.*’ [31*, *M*, *41-50yrs]*
**Hormones**	*‘We kind of thought maybe it was a thyroid problem or something like that*, *didn’t we*?*’ [02*, *M*, *51-60yrs]* *‘When my husband and that kept saying*, *“You’re not getting your words out right*,*” […]I thought it was more my menopause*.*’ [26*, *F*, *41-50yrs]* *‘We put it down to PMT but now I think about it*, *it probably wasn’t all PMT*.*’ [05*, *F*, *41-50yrs]*
**A virus or infection**	*‘I thought I’ll leave it for a few weeks because it could just be something that disappears*, *once the cold is completely out of my system*, *it could just kind of go’ [23*, *F*, *21-30yrs]* *‘I had what I thought was a viral infection back in early October and I noticed that I was slurring my words*, *I thought that was linked to a virus*.*’ [21*, *M*, *51-60yrs]* *‘When I looked up it would send me off balance […] then I thought I had vertigo or a middle ear infection*, *I thought*, *“Oh*, *I’ve got something like that going on*.*”‘ [03*, *F*, *31-40yrs]*
**Infrequent explanations that triggered help-seeking**	
**Stroke**	SON: *‘He was really struggling to do anything with one hand*, *had no strength in it at all*. *I just said to them*, *“I think you need to take him now because it looks like he might have had a stroke’ [16*, *M*, *61-70yrs]* PARTNER: *‘I started trying to make conversation with you and it was just random words and rubbish*, *but then you were talking to the dog okay*. *[…] I said*, *no*, *this isn’t right*, *so I said do the smile*, *lift your arms up*, *walk*, *because I thought*, *stroke […] I called 999*.*’ [28*: *M*, *51-60yrs]*
**Alzheimer’s or Dementia**	*‘I literally thought I had gone cuckoo*, *so I sat here one night and Googled Alzheimer’s and I thought that’s what I’ve got*, *early onset of Alzheimer’s or something*, *I’m depressed*, *I’ve got memory loss*, *my hair’s falling out*, *headaches*. *I thought*, *“That’s it*, *that’s what it is*.*”‘ [03*, *F*, *31-40yrs]* FRIEND: *‘‘One of the things that was really quite frightening and you yourself had concerns over it*, *being early onset dementia*.*’ [26*, *F*, *41-50yrs]* *‘Because I keep forgetting things and because my brain wasn’t working as well as it could I Googled Dementia’ [37*, *F*, *31-40yrs]*
**Problem with eyesight**	SPOUSE: *‘He said*, *“Cor*, *I got a bloomin’ headache*.*” And I said*, *“Please*, *just go to the opticians and get your eyes tested*. *Probably your glasses need changing”*.*’ [19*, *M*, *61-70yrs]* *‘I just thought [my vision is] not quite as good as it has been and therefore… this is time when I actually need to get some glasses*. *I thought obviously increased use of computers and things like that*, *it’s gonna be I need to maybe get some glasses*.*’ [03*, *F*, *31–40]* *‘I was worse than my mate with macular degeneration*, *and he’s got it in both eyes*, *so he should have been worse than me*. *So perhaps that was the turning point when I actually thought there was a problem*.*’ [32*, *M*, *71-80yrs]*

#### ‘Something was wrong’

It was often only if symptoms worsened (reflecting the ‘Rate of change rule’), became more frequent (reflecting the ‘Duration rule’), impacted their day to day activities (reflecting the ‘Severity rule’), or were clearly different from normal (reflecting the ‘Novelty rule’) that patients considered something was not right and may need attention (see Tables 1 and 3). In some circumstances, patients or others just found it difficult to understand the changes. They felt that something was not right and it did not make sense. This uncertainty was often a trigger to visit a HCP, in order to find an explanation.

‘I’d also stumbled a couple of times on site which, you know, I thought that that’s not right as well, it can’t be.’

[08, M, 61-70yrs]

SPOUSE: *‘And you said you didn’t feel right*, *not just that giddiness*, *another day… you was on the settee*, *remember*? *You said*, *“I don’t feel right but I don’t know what’s wrong”*.*’*

[30, M, 71-80yrs]

SPOUSE: ‘*Then it went again and it was a lot more noticeable and lasted a lot longer*. *I said to [PATIENT]*, *“This isn’t right*, *love*, *this shouldn’t be happening”*.*’*

[38, F, 51-60yrs].

‘Yes, I knew things were changing and I couldn’t figure it out. That’s what got me. I couldn’t work it out.’

[33, M, 51-60yrs]

‘I said to [SPOUSE], “I can’t understand why I’m this bad.” So we phoned the GP.’

[32, M, 71-80yrs]

Family members also noted that they knew something was not right if they suggested seeking help from the doctor and the patient agreed without hesitation, especially if the patient would usually be reluctant to seek help.

DAUGHTER: *‘I went up to dad and said*, *“Dad*, *they are going to send the paramedics out*.*” And he went*, *“Good*, *I think I need them*.*” Whereas before if anything like that*, *“What the hell you done that for*? *What are you making a fuss for*?*” So then straightaway then I knew something’s not quite right*.*’*

[19, M, 61-70yrs]

PARTNER: *‘He doesn’t like doctors and I thought he must really be ill to say yes to me*, *you know*.*’*

[17, M, 61-70yrs]

Alongside noting they felt ‘*something was wrong’*, specific explanations linked these deductions to perceived need to seek help (see Table 4). Sometimes changes were attributed to an existing illness or to new or existing mental health problems such as anxiety and depression. Patients (or their family) believed some changes were caused by hormones, such as thyroid problems, the menopause, premenstrual tension or other changes associated with the menstrual cycle. This often meant the patient did not feel a need to visit a HCP, or not do so urgently, especially if attributed to an existing problem (reflecting the ‘Novelty rule’, see Tables 1 and 3) even though they thought something was wrong.

There were relatively few instances where a patient (or their family) believed they were experiencing symptoms of a new medical condition or illness. If patients experienced slurred speech, or weakness in a body part, this was sometimes attributed to a stroke and prompted help-seeking. Change in cognitive function sometimes triggered concerns of Alzheimer’s disease or dementia. Changing vision, problems reading, and headaches made some people think they had problems with their eyesight and needed to visit an optician to get their eyes tested or get new glasses (see Table 4).

#### The social context of symptom appraisal

Symptom appraisal occurred within the social context. People spoke to friends, family and work colleagues about some of the changes they were experiencing, actively asking for advice, and this helped them to develop explanations for changes. Sometimes, other people enquired about symptoms or changes they had noticed. Not everything was shared, however. A few patients kept changes in cognitive function or concerns about those changes from others.

‘She [was] asking me what I was feeling and stuff, because you go really pale afterwards, and she said, “I don’t know what’s going on.” […] she just went on her phone, and she’s going, “Well, I’ve got it all up here, NHS, and it’s saying….’

[29, F, 41-50yrs]

‘I spoke to someone about it and said, “I keep getting this feeling in my hand.” They mentioned that a friend of theirs had had carpel tunnel syndrome, and that sort of made a bit of sense, like spasms in your hand.’

[33, M, 51-60yrs]

MOTHER: *‘You never discussed those things with us did you*? *[…] about your feelings if you were getting Alzheimer’s and things like that*.*’* PATIENT: *‘No*, *because it’s embarrassing*, *it’s terribly embarrassing’*.

[03, F, 31-40yrs]

When family, friends and colleagues were aware of changes they often encouraged a patient to seek help and this resulted in a joint decision to visit a GP or other HCP.

‘I had four people come up to me, deeply concerned about my behaviour, basically. They just said I’m not right. Also my partner mentioned that I was stammering a little bit; my speech wasn’t right […] One [friend] who plays golf occasionally [with me], and he says, “Look, you go and see your GP, I’m a bit concerned about you”, and that’s what I done.’

[09, M, 61-70yrs]

‘He made me go and do it, ‘cause he just said,” this isn’t right, you shouldn’t be waking up every morning and taking these pills and then keep on taking them”.’

[05, F, 41-50yrs]

Typically, the explanation for changes was shared between the patient and others, although sometimes there was disagreement and sometimes patients did not agree they needed care.

‘She knew there was something wrong and she kept saying every day and me being awkward, saying, “No, its fine. It’s fine,” and she would go on every day to me.’

[33, M, 51-60yrs]

‘Because I was always on the sofa asleep and he used to joke and say “are you depressed because that is what depressed people do, sleep all the time.” And I used to say, “no I’m okay I’m just tired, I’m just really tired”.’

[13, F, 31-40yrs]

MOTHER: *‘I said to her*, *“If you’re not happy and it’s still there you must go back*.*” She was like*, *“I don’t want to go back to the doctors*.*” It was a typical*, *“I don’t want to go*.*”*

[03, F, 31-40yrs]

‘He said about going to the GP, a couple of times actually. and I’m like, “Oh no, I can’t be bothered. It’s only something small. It goes with paracetamol.”

[39, F, 21-30yrs]

In some circumstances, the decision to seek help was made by others, either because the patient had diminished capacity to make that decision, or because it was deemed a medical emergency. Four of the five patients who consulted a HCP within a week did so as someone had called an ambulance on their behalf.

SON: *‘I just said to them*, *“I think you need to take him now because it looks like he might have had a stroke at some point in the last few days or week*,*”*.*’*

[16, M, 61-70yrs]

She took one look at me and she said, you’re not well, you’ve had a stroke. […] she said, I’m taking charge of this, bossy cow, rang 111 ambulance paramedics what have you, […] into the stroke unit

[27, F, 71-80yrs]

### The Help-seeking interval: Barriers to seeking help

This section focuses on barriers to seeking help once a person or their family had considered consulting a HCP. It outlines processes and anticipatory factors that influenced behaviour occurring within the help-seeking interval. The sub-themes concern the most prominent barriers: outcome expectations, impediments of the healthcare system, and proximal goals which lessened the urgency with which care was sought.

#### Outcome expectations about seeking help

Anticipation of what would happen upon help-seeking put some people off visiting their GP or other HCP. Sometimes these outcome expectations were physical. For instance, they were unconvinced that visiting a HCP would be helpful in resolving the symptoms.

SPOUSE: *‘If I’d have gone to a doctor I don’t think they would have actually taken too much notice of me’*

[20, M, 71-80yrs]

FRIEND: *‘You yourself had concerns over it being early onset dementia […] And it was really quite frightening to think if that’s where it’s headed and we’re pushing towards a diagnosis*, *that’s it*, *you know*, *there’s nothing you can do about it*. *There’s only so much medication*.*’*

[26, F, 41-50yrs]

‘Going to see a doctor, you think you’re wasting the time, because they’ll say, “Oh, it’s just something that is part of you,” or, “Don’t worry about it”.’

[29, F, 41-50yrs]

Other outcome expectations that acted as barriers to seeking help were concerned with judgements that they thought other people would make. Participants spoke about not wanting to waste a GP’s time if the problem turned out to be nothing serious, especially if they were ambivalent about what it was or when they had to re-visit about a continuing problem.

‘We’re told all the time that, you know, don’t waste your GP’s time going to them over a virus or flu because they are not going to respond with antibiotics or whatever, and the best help is to self-medicate […] and that is what I did.’

[21, M, 51-60yrs]

‘I mean, the doctors are so busy now, you don’t want to keep bothering them with every little thing because there’s people that need to see the doctors and can’t get in. It’s just a funny thing to explain to a doctor, that you get déjà vu and then a smell.’

[29, F, 41-50yrs]

Some patients noted they ‘felt silly’ about seeking help, either because they might be wrong or they were concerned about what the doctor would think of them–they did not want to be seen as a hypochondriac, visiting unnecessarily, or too often. For some this was a social outcome expectation centred on concerns about what other people may think. For others, it was a self-evaluative outcome expectation—they did not want to be someone who consults a GP at every opportunity and thus were reluctant to visit or revisit the doctor.

‘I didn’t want to go and say to him, “I think I’ve got Alzheimer’s.” He’d think I was silly because I was [31–40] at the time, so he’s probably going to think, “Oh gosh, she’s a hypochondriac”.’

[03, F, 31-40yrs]

‘I took the amitriptyline actually for a week, because you don’t want to keep going to the doctor’s for a headache, well, I just didn’t want to anyway. […]. She said to come back in a couple of days if it didn’t work, but I waited a week, because I kept feeling silly.’

[24, F, 41-50yrs]

MOTHER: *‘You were actually half thinking you were being a bit silly going back again didn’t you*, *but you just thought well I’ll get some migraine tablets*.*’*

[36, F, 21-30yrs]

‘Part of it was my fault for not going back and saying, “Look, these headaches haven’t changed I need to do something. What are you going to do for me?” But I was never one for going to the doctors and complaining about anything.’

[05, F, 41-50yrs]

‘I probably should have done but I’m not inclined to be a ‘mentioner’ of such things.

[07, F, 31-40yrs]

#### Impediments of the healthcare system

Participants spoke about the difficulty in accessing prompt care, especially when they did not believe they had a medical emergency. In particular, participants noted that they had wait for up to 3 weeks for an appointment with their GP or had to visit A&E instead.

FRIEND: ‘*You had to wait three weeks for an appointment to see the GP*.*’*

[26, F, 41-50yrs]

‘Cos my surgery was brilliant; I could make an appointment now and I would see them at 5 o’clock. Now I have to wait 2–3 weeks unless it’s urgent.’

[09, M, 61-70yrs]

‘If you want a doctor wait 2 or 3 weeks. With most people by the time you’ve waited that you’re better or you’ve gone to the hospital, as we did, to get attention that day. So yeah, it’s a very slow process.’

[18, F, 61-70yrs]

Participants also noted that their preference for a specific doctor, due to wanting continuity of care or a better doctor-patient relationship, led some people to postpone seeking care until their preferred doctor was available.

‘I made that appointment, … I made it and it was quite a lot later, I wanted to see this doctor because I like her and I had already seen her.’

[07, F, 31-40yrs]

‘She just said, “Oh, can you make sure you come back?” And then I just thought, “I really don’t like you”, (laughter) I just didn’t like her at all. And that’s when I went back again…I don’t know when that was, that was a good few weeks though, to see this other lady.’

[26, F, 41-50yrs]

‘Every time I try and see a different doctor they go, “Right, so we need to go through your entire history” and I’m like, “What? We’re going to be here all day!” … I have to go through everything again and it’s just draining and I’m like no, I’d just rather see the doctor who knows what’s going on with me.’

[06, F, 31-40yrs]

#### Proximal goals

Some people did not discuss their changes/symptoms with a HCP as they had other more pressing priorities such as childcare or other comorbidities to deal with: they had proximal goals that required more immediate attention.

‘I didn’t have time to think about [going to the GP] because my mother was dying, my son had gone off the rails a bit, and my daughter had anxiety problems, so there were things going on, but even if I had noticed them, I don’t know if I would have gone’

[24, F, 41-50yrs]

‘I was having a moan about my back, not getting any sleep because I can’t sleep in the night because I’m in pain, so all the time about my back and about my legs and whatever else, and I always kept forgetting to mention my ear.’

[06, F, 31-40yrs]

FRIEND: You’re a working mum and you tend to just get on with it. […] It’s not about you, is it? […] As far as you’re concerned, you just think, “okay, headache, whatever, I’ve got to do this today, so I can’t”.

[26, F, 41-50yrs]

## Discussion

This study has revealed that for people recently diagnosed with brain cancer, changes and symptoms are often noticed many months before presentation. The in-depth interviews uncovered the thoughts and reactions to these changes and how this shifted throughout the pathways to diagnosis. A strength of this study is that the sample was diverse and patients were interviewed within a few weeks of their diagnosis and prior to neuro-surgery, facilitating recall [23]. They were also encouraged to have a family member present during the interview. Both approaches reduced the potential for recall bias and provided a fuller picture of the pathway to diagnosis. Furthermore, the workshop for GPs, and other patients and their family members, gave us an opportunity to triangulate our analyses and check the credibility of our findings. The main limitation was that the participants were often unwell, and sometimes apprehensive about their imminent major surgery. Whilst no obvious differences were apparent, it is possible that there were differences between the ‘public’ accounts given in the interviews, often in front of loved ones, and participants’ actual views. It is difficult to know if this was the case without conducting further interviews. Our purposive sampling enabled a range of perspectives to be heard. There may have been differences in experience and accounts between those of differing age and sex. Future quantitative work could explore this.

Participants described experiencing multiple, subtle changes rather than symptoms. In addition to bodily changes, subtle changes included loss of interest, less engagement or less ability to engage with daily living activities. For some this was caused by a symptom (e.g. problems changing gear believed to be due to weakness in arm). For others the changes appeared unconnected to physical changes and was not seen as a health issue at all (e.g. problem changing gear, believed to be due to a problem with the car). The occurrence and attribution of non-somatic changes is currently missing from theoretical models and frameworks used to understand symptom appraisal and help-seeking behaviour as they state the patient has ‘signs or symptoms’ [24,25], a ‘physical state’ [26], or a ‘bodily change’ [13]. Campaigns to encourage timely presentation, research into help-seeking behaviour, and the theoretical models underpinning this research would benefit from considering an expansion to include non-somatic changes. Bringing non-somatic changes into awareness campaigns may be problematic. For instance, it is as odds with other campaigns aimed at reducing GP attendances for minor ailments. However, for brain cancer, a combination of seemingly trivial changes may represent the first opportunity for a timely diagnosis.

As highlighted for other cancers [27–29], the subtlety and normality of changes often led participants to believe there was nothing wrong. Often it was only if something altered (e.g. worsened, became more frequent, noticeably interfered with life, or was different from normal) that participants started to think something was not right and considered the need for care. However, some attributions of changes were not reappraised until after diagnosis. Patients with multiple changes/symptoms had ultimately sought help for some of these symptoms and this had resulted in diagnosis. Only with hindsight had they come to realise that other changes they had experienced, and dismissed or normalised, may have also been due to the brain cancer. This experience of multiple subtle changes, plus some people’s selective disclosure to HCPs, could be developed as targets for initiatives in primary care. For instance, ensuring GPs are aware of the range of changes beyond a headache, seizure and one-sided weakness, and that many changes may be unreported. Therefore vague symptoms, including ‘just not yourself’, need thorough exploration by family doctors and a good history should be taken from patients. Patients could be also encouraged to bring written lists of changes/ symptoms and consider the possibility that changes are linked, rather than only reporting the single symptom/change that concerns them most. Inclusion of family and friends may also be important as patients may not notice all the symptoms themselves. This is important as friends and family often helped patients reach an explanation for their changes/symptoms and were involved in the decision to seek and re-seek help. Policies limiting the number of symptoms per appointment [30] and short consultations may be a hindrance, as such appointments are not sufficient to share subtle, intermittent changes/symptoms and could contribute to selective or limited disclosure. However primary care HCPs often still have the benefit of continuity of care, and may be in a position to also identify unusual patterns in specific patient groups. Initiatives in primary care could be an extension to the ‘HeadSmart: Be Brain Tumour Aware’ awareness campaign. This was launched in the UK in 2011, and provided guidance on symptom awareness, assessment, investigation and referral with regard to paediatric brain cancers. The campaign enhanced awareness among health professionals and the public, and appears to have led to a significant reduction in the UK’s total diagnostic interval [31].

Using the CSM and SCT to underpin the analysis provided a useful way of capturing the processes leading up to consultation with a HCP. What the patient or family thought about the changes influenced their responses, in line with the CSM. The most common explanations included tiredness, stress, age or recent events, and these were rarely believed to need HCP attention (in line with ‘Stress-illness’, ‘Age-illness’, and ‘Chronology’ rules). People made these inferences because the changes they were experiencing were infrequent, slight, common or rarely obstructed functions, in line with the ‘Duration’, ‘Pattern’, ‘Prevalence’, and ‘Severity’ rules respectively. Cognitive heuristics also influenced re-appraisal of changes/symptoms leading participants to believe something was not right. Thus, the cognitive heuristics of symptom appraisal appear to play a crucial role the decision to seek help. However, despite their inclusion in the much-used CSM, they have lacked attention and are considerably under-researched. Our focus group participants called for increased public awareness of brain cancer and the potential symptoms. However, given the changes are numerous, subtle and common, it may be difficult to achieve significant change by raising awareness. Campaigns could tackle cognitive biases rather than solely promoting symptom lists. For instance, this could involve raising awareness of the biases and asking people to question themselves if putting symptoms down to ageing or stress as a matter of course. Myth-busting messages could highlight that even symptoms that are intermittent or mild or common can be signs of a serious health problem, and advise people not to wait until symptoms get worse or start interfering with everyday life. As these messages could be applicable to other cancers, this might also be a more cost-effective approach. Again there is the challenge of not overloading health services with those with minor ailments. There will be a trade-off between increased diagnostic pick-up and ‘unnecessary’ attendances. Currently the threshold does not favour early diagnosis and may need to be reconsidered. It is worth noting here that the role of early diagnosis of brain cancer on improving survival is currently unclear. However, early diagnosis has the potential for providing symptom relief and improving the acceptability of the patient journey and psychological wellbeing [32].

An additional trigger to consider seeking help was uncertainty. Changes/symptoms that could not be easily explained or understood by patients or their family were deemed to warrant the attention of a HCP. Within the CSM this could be considered as the coherence aspect of illness representations. Moss-Morris et al., [33] describe illness coherence as a meta-cognition reflecting how the illness (or in this case symptom) ‘‘makes sense” as a whole to the patient and may play an important role in adjustment and response to symptoms. Again, this has implications for interventions to reduce time to presentation. Numerous campaigns have used the slogan ‘*If in doubt get it checked out’* [34, 35]. We found in this study that those in doubt or uncertain about their symptoms did seek help. It was those who were sure it was nothing to be concerned about who waited.

Worry about wasting a doctor’s time has received recent interest after being noted as a factor that could influence future help-seeking for cancer symptoms [36–38], especially in the UK’s government-funded healthcare system. In the current study we found that this outcome expectation was an important anticipatory factor influencing the decision to seek help. This study also highlighted that, in addition participants sometimes ‘felt silly’ for seeking help for possibly trivial symptoms, and lacked confidence in their suspicions that something was ‘not right’. Safety netting (for instance booking follow-up appointments before a patient leaves the surgery or giving a time limit for symptoms to resolve) could help to legitimise healthcare use and could lessen these barriers. Further barriers included issues of access to care (including waiting times and desire to see a specific doctor), and competing priorities. These factors have been shown to be crucial to help-seeking for a range of cancers and other diseases [11,39]. Further work could identify which factors have the greatest magnitude of effect on time to presentation, and whether this differs between initial time to presentation and subsequent time to presentation when a person is deciding whether or not to re-consult. Further work could also investigate the interplay between symptom appraisal and barriers to seeking help. For instance, when people believed something was wrong and definitely in need of care (e.g. a stroke) this in itself lessened the barriers to seeking help. Barriers to seeking help may be more of an impediment when there is less certainty in the need for care and when the need for care is not perceived as urgent.

In conclusion, this is the first study to report the patient perspective of the appraisal and help-seeking intervals in the pathway to diagnosis of brain cancer. Application of psychological theory facilitated understanding of the influences on cognition and behaviour. The study highlights implications for theory and potential opportunities to develop interventions at policy and clinical levels aiming for more timely diagnosis of brain cancer. Education of patients, clinicians and policy makers may help drive improvement in the first instance, with more concrete improvement measures added once further validation studies have taken place.

## Supporting information

S1 File Interview topic guide. (PDF)

S2 File Comparison between interviews and workshops. (DOCX)

S3 File COREQ checklist. (DOCX)

## References

[1] Burnet NG, Jefferies SJ, Benson RJ, et al Years of life lost (YLL) from cancer is an important measure of population burden and should be considered when allocating research funds. *Br J Cancer* 2005; 92(2): 241–245. 10.1038/sj.bjc.6602321 15655548 PMC2361853

[2] Kirkby NF, Jefferies SJ, Jena R, Burnet NG. A mathematical model of the treatment and survival of patients with high-grade brain tumours. *J Theor Biol*. 2007; 245(1): 112–24. 10.1016/j.jtbi.2006.09.007 17084863

[3] Penfold C, Joannides AJ, Bell J, Walter FM. Diagnosing adult primary brain tumours: can we do better? *Br J Gen Pract*. 2017; 67(659): 278–279. 10.3399/bjgp17X691277 28546414 PMC5442949

[4] House of Commons Petitions Committee. *Funding for research into brain tumours*: *first report of session 2015–16* *HC 554**London*: *TSO*, *2016* https://www.publications.parliament.uk/pa/cm201516/cmselect/cmpetitions/554/554.pdf [Accessed September 2018]

[5] National Cancer Research Institute. *Cancer Research Database*: *Spend by research category and disease site* https://www.ncri.org.uk/ncri-cancer-research-database/spend-by-research-category-and-disease-site/ [Accessed September 2018]

[6] Elliss-Brookes L, McPhail S, Ives A, et al Routes to diagnosis for cancer—determining the patient journey using multiple routine data sets. *Br J Cancer*. 2012; 107(8): 1220–6. 10.1038/bjc.2012.408 22996611 PMC3494426

[7] Lyratzopoulos G, Neal RD, Barbiere JM, et al Variation in number of general practitioner consultations before hospital referral for cancer: findings from the 2010 National Cancer Patient Experience Survey in England. *Lancet Oncol* 2012; 13(4): 353–365. 10.1016/S1470-2045(12)70041-4 22365494

[8] Salander P, Bergenheim AT, Hamberg K, Henriksson R. Pathways from symptoms to medical care: a descriptive study of symptom development and obstacles to early diagnosis in brain tumour patients. *Fam Pract* 1999; 16(2): 143–148. 10381020 10.1093/fampra/16.2.143

[9] Davies E, Clarke C. Early symptoms of brain tumours. Journal of Neurology, *Neurosurgery & Psychiatry* 2004; 75: 1205–1206. 10.1136/jnnp.2003.033308 PMC1739177 15258238

[10] Walter FM, Penfold C, Joannides A, Saji S, Johnson M, Watts C, Brodbelt A, Jenkinson MD, Price S, Hamilton W, Scott SE. Missed opportunities for diagnosing brain tumours in primary care? Qualitative study findings. *British Journal of General Practice*. *In press* 10.3399/bjgp19X701861 PMC6428480 30858332

[11] Smith L. K., Pope C., & Botha J. L. Patients’ help-seeking experiences and delay in cancer presentation: a qualitative synthesis. *Lancet*, 2005; 366(9488), 825–831. 10.1016/S0140-6736(05)67030-4 16139657

[12] Walter F.M., Webster A., Scott S.E., & Emery J. The Andersen Model of Total Patient Delay: a systematic review of its application in cancer diagnosis. *Journal of Health Services Research & Policy*. 2012; (2): 110–8. 10.1258/jhsrp.2011.010113 PMC3336942 22008712

[13] Scott SE, Walter FM, Webster A, Sutton S, Emery J. The model of pathways to treatment: conceptualization and integration with existing theory. *Br J Health Psychol*. 2013; 18(1): 45–65. 10.1111/j.2044-8287.2012.02077.x 22536840

[14] Lyratzopoulos G, Saunders CL, Abel GA, McPhail S, Neal RD, Wardle J, Rubin GP. The relative length of the patient and the primary care interval in patients with 28 common and rarer cancers. *Br J Cancer*. 2015; 31;112 Suppl 1: S35–40. 25734380 10.1038/bjc.2015.40 PMC4385974

[15] Leventhal H, Meyer D, Nerenz DR. (1980). The common-sense model of illness danger In Rachman S (Ed.) *Medical Psychology (Vol II)*. New York: Pergamon Press.

[16] Bandura A. *Self-efficacy*: *The exercise of control*. 1997; New York: Freeman.

[17] Bandura A. Health promotion from the perspective of social cognitive theory. *Psychology & Health*, 1998; 13(4): 623–649.

[18] Cacioppo J.T. Andersen B.L., Turnquist D.C. & Petty R.E. Psychophysiological comparison processes: Interpreting cancer symptoms In Andersen B.L. (Ed.) *Women with cancer*: *Psychological perspectives* (pp.141–171). 1986; New York: Springer-Verlag.

[19] Leventhal H, Brisette I, Leventhal EA. The common-sense model of self-regulation of health and illness In Cameron L.D & Leventhal H (Eds.) *The Self-Regulation of health and Illness Behaviour* (pp 41–65). 2003; New York; Routledge.

[20] Tong A, Sainsbury P, Craig J. Consolidated criteria for reporting qualitative research (COREQ): a 32-item checklist for interviews and focus groups. *International Journal for Quality in Health Care*. 2007; 19(6): 349–357. 10.1093/intqhc/mzm042 17872937

[21] Braun V CV. Using thematic analysis in psychology. *Qual Res Psychol*. 2006; 3:77–101.

[22] Gale NK, Heath G, Cameron E, Rashid S, Redwood S. Using the framework method for the analysis of qualitative data in multi-disciplinary health research. *BMC Med Res Methodol*. 2013; 13: 117 10.1186/1471-2288-13-117 24047204 PMC3848812

[23] Weller D., Vedsted P., Rubin G. et al, The Aarhus statement: improving design and reporting of studies on early cancer diagnosis. *Br J Cancer* 2012; 106(7):1262–7. 10.1038/bjc.2012.68 22415239 PMC3314787

[24] Andersen B. L., Cacioppo J. T., & Roberts D. Delay in seeking a cancer diagnosis: Delay stages and psychophysiological comparison processes. *British Journal of Social Psychology* 1995; 34(1), 33–52. 7735731 10.1111/j.2044-8309.1995.tb01047.x

[25] Cacioppo J.T. Andersen B.L., Turnquist D.C. & Petty R.E. (1986). Psychophysiological comparison processes: Interpreting cancer symptoms In Andersen B.L. (Ed.) *Women with cancer*: *Psychological perspectives* (pp.141–171). New York: Springer-Verlag.

[26] Cioffi D. Beyond attentional strategies: A cognitive-perceptual model of somatic information. *Psychological Bulletin* 1991; 109: 25–42 2006227 10.1037/0033-2909.109.1.25

[27] Emery J.D., Walter F.M., Gray V. et al Diagnosing cancer in the bush: a mixed-methods study of symptom appraisal and help-seeking behaviour in people with cancer from rural Western Australia. *Family Practice* 2013; 30: 294–301. 10.1093/fampra/cms087 23363540

[28] Evans R.E., Morris M., Sekhon M., et al (2014). Increasing awareness of gynaecological cancer symptoms: a GP perspective. *British Journal of General Practice* *2014;* 64: e372–80. 10.3399/bjgp14X680161 24868075 PMC4032020

[29] de Nooijer J., Lechner L., de Vries H. A qualitative study on detecting cancer symptoms and seeking medical help; an application of Andersen’s model of total patient delay. *Patient Education and Counseling* 2001; 42(2): 145–157. 11118780 10.1016/s0738-3991(00)00104-x

[30] McCartney M. One problem. *BMJ* 2014; 348: g3584 10.1136/bmj.g3584 24888509

[31] HeadSmart: Be Brain Tumour Aware. A new clinical guideline from the Royal College of Paediatrics and Child Health with a national awareness campaign accelerates brain tumor diagnosis in UK children—“HeadSmart: Be Brain Tumour Aware.” *Neuro-Oncology*. 2016;18(3):445–454. 10.1093/neuonc/nov187 26523066 PMC4767243

[32] Hamilton W, Stapeley S, Campbell C, Lyratzopoulos G, Rubin G, Neal RD. For which cancers might patients benefit most from expedited symptomatic diagnosis? Construction of a ranking order by a modified Delphi technique. BMC Cancer; 2015; 15:820 10.1186/s12885-015-1865-x 26514369 PMC4627396

[33] Moss-Morris R., Weinman J., Petrie K., Horne R., Cameron L., Buick D. The Revised Illness Perception Questionnaire (IPQ-R), *Psychology & Health* 2002; 17: 1–16,

[34] If in doubt, get checked out. *BDJ* 2006; 201: 694

[35] British Association of Dermatologists. (2013). http://www.bad.org.uk/shared/get-file.ashx?id=3913&itemtype=document [Accessed October 2018]

[36] Forbes L.J., Simon A.E., Warburton F., et al Differences in cancer awareness and beliefs between Australia, Canada, Denmark, Norway, Sweden and the UK (the International Cancer Benchmarking Partnership): do they contribute to differences in cancer survival? *BJC* 2013; 108; 292–300. 10.1038/bjc.2012.542 23370208 PMC3566814

[37] Cromme S.K., Whitaker K.L., Winstanley K., et al Worrying about wasting GP time as a barrier to help-seeking: a community-based, qualitative study. *British Journal of General Practice* 2016; 66: e474–82. 10.3399/bjgp16X685621 27215569 PMC4917050

[38] Hall N., Birt L., Banks J., et al Symptom appraisal and healthcare-seeking for symptoms suggestive of colorectal cancer: a qualitative study. *BMJ Open* 2015; 5: e008448 10.1136/bmjopen-2015-008448 26453591 PMC4606388

[39] Taber J.M., Leyva B., Persoskie A. Why do people avoid medical care? A qualitative study using national data. *Journal of General Internal Medicine* 2015; 30: 290–7. 10.1007/s11606-014-3089-1 25387439 PMC4351276

