# Supplementary material for: ‘It was nothing that you would think was anything’: Qualitative analysis of appraisal and help seeking preceding brain cancer diagnosis
Source: PLoS One. 2019 Mar 22;14(3):e0213599. doi: 10.1371/journal.pone.0213599 (PMC6430370; doi:10.1371/journal.pone.0213599)
Supplement: S1 File — (PDF) [file pone.0213599.s001.pdf]

Interview Topic Guide

Note on use of topic guide

As this is an exploratory study, we wish to encourage participants to discuss their experiences and views in an open way without excluding issues which may be of importance to individual participants and the study as a whole. Therefore, unlike a survey questionnaire or semi-structured interview, the open questions will be formulated by the interviewer, and questioning (and the language and terminology used) will be responsive to respondents' own experiences, attitudes and circumstances.

The following guide does not contain pre-set questions but rather lists the key topics and sub-topics to be explored with each participant. Topics for exploration are underpinned by Walter's model of Pathways to Treatment[30-31]. Topics do not include follow-up questions like `why', `when', `how', etc. as participants' contributions will be fully explored throughout using prompts and probes in order to understand how and why views, behaviours and experiences have arisen. The order in which topics are addressed will be flexible, and the amount of time spent exploring different topics, will vary between participants according to individual demographics, clinical factors and circumstances.

1. Welcome & introduction
   - Introduce self & PCU, DPHPC/UoC
   - Introduction to research: commissioned by The Brain Tumour Charity to improve understanding and develop ways of diagnosing brain cancer early
   - Reason for asking them to participate
   - Discuss confidentiality and anonymity
   - Use of digital recorder and data storage
   - Length of interview and nature of discussion (specific topics to cover but will be like a conversation, 'there are no right or wrong answers, in your own words')
   - Any questions?

2. Consent (confirm understanding of study and consent participant)

3. Background

    a. Open with general background questions to obtain socio-demographic data: age, marital status, living arrangements, education and employment history, daily activities, hobbies, general health etc.
    b. Explore participant's illness experience to obtain broad narrative account from first thing they noticed that was different through to diagnosis
    c. Explore participant's confidence and feelings about interacting with healthcare providers and obtaining and understanding medical information (e.g. making sure HCPs understand their problems, ability to discuss concerns with HCP, ability to obtain and understand information and ask questions etc.)

4. Appraisal interval

Explore events, processes and contributing factors associated with appraisal interval in detail

    a. Obtain detailed description of symptom(s) and how experienced (prompt for any other symptoms noticed)
    b. Explore how symptom(s) were noticed/recognised (e.g. by self, family member, friends, colleagues, incidentally by HCP)
    c. Explore any discussion of symptoms with others (with whom, nature of discussion, any advice offered)
    d. Explore participant's understanding of (interpretation and attribution) symptoms and thoughts about what might be wrong
    e. Explore any information-seeking in relation to symptoms (e.g. internet)
    f. Explore participant's feelings and emotions in relation to symptom(s)
    g. Explore coping mechanisms and self-management (including practical and emotional)
    h. Discuss whether and how symptom(s) changed, developed, progressed
    i. Discuss any nondisclosure of symptoms and reasons for this
    j. Explore any previous experience of brain tumours / brain cancer and impact of this
    k. Introduce calendar landmarking tool and draw timeline of each symptom
    l. Check for any other symptoms not spontaneously discussed, prompt for symptoms related to:
        i. impairment of normal brain function (focal neurological deficit) – including:
            1. physical symptoms (any changes to how participant's body works)
            2. emotional and behavioural symptoms (any changes in how participant feels, or personality and behaviour)
            3. cognitive symptoms (any changes or problems with thinking, reasoning or memory)
        ii. raised/increased intracranial pressure (headache, nausea/vomiting, seizures, changes in vision, drowsiness, confusion and irritability, dizziness, balance problems)
    m. Explore anything else that was unusual or out of character (whether or not considered related to diagnosis)

5. Help-seeking interval (for participants who attended GP prior to diagnosis or emergency presentation)

Explore events, processes and contributing factors associated with help-seeking interval

   a. Explore timing of decision to seek medical advice – reasons (e.g. a specific event or trigger) and any involvement of other people (e.g. family members, friends, colleagues); including any reasons for delaying seeking medical advice
   b. Explore first GP appointment when symptoms were discussed, including expectations about what might happen at appointment, and nature of discussion (including whether all symptoms were discussed or not and why)
   c. Explore outcome of first appointment (including any referrals/tests/investigations and information provided about these)
   d. Explore feelings about outcome of first appointment
   e. Explore any subsequent appointments with GP – explore reasons for subsequent appointments (trigger for visit including safety netting, symptoms discussed, outcomes etc.)
   f. Explore feelings about subsequent GP appointments
   g. Explore any referrals/tests/investigations discussed at these appointments and information provided about these
   h. Explore any re-appraisal interval (explore with participants who cycled back and forth between appraisal and help-seeking intervals)
   i. Use calendar landmarking tool to date first and subsequent GP or other healthcare appointments / referral to secondary care / emergency presentation and review/revise symptom timelines

6. Diagnostic interval

Explore events, processes and contributing factors associated with diagnostic interval

   a. Explore participant's understanding of reason for referral and what it involved
   b. Explore referral (if applicable) and experience of referral and outcome of appointment(s)
   c. Explore any emergency presentation with symptoms – details of what happened and experience and understanding of outcome
   d. Fully explore any delays (patient, health system factors, clinical factors etc.) from patient's perspective
   e. Use calendar landmarking tool to date diagnostic interval

7. Ask whether anything else participant would like to mention / discuss

8. Thank participant and close interview (confirm consent and confidentiality, ask whether would like to be contacted about stakeholder workshop, highlight sources of support detailed on Participant Information Sheet)
