# Supplementary material for: ‘It was nothing that you would think was anything’: Qualitative analysis of appraisal and help seeking preceding brain cancer diagnosis
Source: PLoS One. 2019 Mar 22;14(3):e0213599. doi: 10.1371/journal.pone.0213599 (PMC6430370; doi:10.1371/journal.pone.0213599)
Supplement: S2 File — (DOCX) [file pone.0213599.s002.docx]

**S2 Supporting Information. Comparison between interviews and workshops**

| **PATIENT AND RELATIVE INTERVIEWS** | **PATIENT, CARER & GP WORKSHOP** |
| --- | --- |
| ***1.0 APPRAISAL INTERVAL*** | |
| ***1.1 Subtlety and normality of changes: I didn’t think anything of it*** | |
| Slight, subtle changes (‘Pattern rule’) | Subtle  - - G1.1. p4 by patient, and by GP (“I think a lot of it is very subtle signs”)   - “very subtle change” female GP, g1.1. p7   - G1.2 p13 “The symptoms are very vague” by male GP and female GP   - R8 G2.2 p21. Relative. “Not big cognitive changes” Pt says the changes were subtle. R1 said “it was very subtle and over maybe 3, 4 weeks”  Trivial  - - “I didn’t really put it to much” R4 G2.1 p14. Patient   - “I didn’t really put that down to anything”. Relative   - Pt collapsed and his wife thought “Oh it’s only fainting” which has happened before. By *relative*   - G2.2 p10. R1 “You don’t want to go and think it’s something trivial, so you don’t want to bother”. Relative.   - R2 G2.2 p22 “Thinking you’re in the wrong lane, you’d never stop to think that” .. “my wife must have something seriously wrong with her brain”. Relative.   - G2.2 p12 “Just try and trivialise it because we just want to keep calm and carry on”. By R4 (GP)   - R2 G2.2. p12. Patient: “It is … part of being British… let’s do this the right way and chin up and not bother the doctor”.   - G2.2. p11 R7. Pt initially thought the symptoms were “strange” but “carried on working”. By patient. |
| Numerous alterative and more likely explanations. | Non-specific  - - Symptoms are generalised. R2 (patient) G2.2. p20: “Because you’re getting symptoms elsewhere in your body, like the twitching eye or the numbness, you’re not always going to suddenly think, it’s got to be a brain tumour” whereas “people who… say about “I think I’ve got a brain tumour” are the ones … with headaches”   - *Relative* makes a comparison with his asthma diagnosis. R1 G2.2. p20 talks about how it took 18 months to eventually get an asthma diagnosis after multiple coughing, but during that time, the diagnosis could have been anything! “that’s the difficult, I think, because it’s non-specific”.  Patients do not think it could happen to them.  - ““No, I can’t possibly lose my mother and my wife, that’s so ridiculous,” by relative who didn’t think there could be anything wrong with his wife because he has recently lost his mother. G1.1 p3” - R6 G2.2. p12 “I haven’t because I don’t want to think that it’s going to happen to me”- Pt talks about not getting himself checked for Alzheimer’s when he forgets something - R2 G2.2 p20 says that both pt and partner never thought they would both get brain tumours. |
| Changes infrequent and intermittent (‘Duration rule’ ) | Infrequent  - - G1.1. p4 by relative “they are so subtle and so infrequent” |
| little interference with work and other responsibilities (‘Severity rule’) |  |
| Normalisation- did not warrant attention of HCP | Common  - - Not every symptom is bad. R10 G2.1 P12, GP: A lot of people have “normal symptoms” at any one point e.g “at any one point in time, ¾ of the population will have a symptom”   - Normalisation of symptoms by others e.g. G2.1 p2 *relative* other people say “yeah, I have that same problem”   - “how common pins and needles are and can be a common symptom or tiredness”. *GP* R10 G2.1 p21.   - *GP,* G2.2 p17 R5 “Half the population feel tired, so it’s… so common” and “actually a lot of it is stress from people burning out”.     - Work and exhaustion is very common. R5 G2.2. p17 and R1     - Need to differentiate tiredness due to work and tired “on weekends” or on holiday. R5 G2.2. p17. |
| *Common explanations unlikely to prompt help-seeking (*stress, busy at work, tiredness, age, recent events) | *What stops patients presenting earlier?*   - Normal stress and aging   - Easier to blame social situations such as age, and personality change triggered by environment   - G2.2. p12 *relative:* “But that’s it with memory, we all suffer from it, especially as you get older”.   - G2.2 p21 *relative:* “I didn’t think anything of it other than the fact that she was working long hours, so she was tired” - G1.2 p10, *relative* says wife’s symptoms were attributed to jetlag and becoming an “empty nester” and “it disguised a lot of those symptoms”  Normal old age  - - - G1.2 p13 and p14     - G2.2. p12 “But that’s it with memory, we all suffer from it, especially as you get older”. *Relative.*  Wisdom teeth  - - - G1.1. p6 by patient ““It might be your wisdom teeth. It might be this, that or the other.” And I’m like, “No, I got a fuzzy sensation here.””  Hangover G2.1 p8 by patient. even though pt remembers not drinking. “I kept on feeling like I had a hangover when I know I hadn’t had a drink. So I kept on thinking, why do I feel sluggish? Why does my head feel fuggy?”   - Pt colluded with herself in telling herself that the symptoms are like a hangover, even though she has not been drinking the previous night.  Stress  - - - “I collude with them” with regard to pt being busy. Said by GP R3 G2.1 p8  “I think a lot of patients will say something, and will go, as we’ve all heard before, “I’m stressed, I’ve got lots going on at work,” and you both kind of sort of collude a little bit and think maybe that is the case but what else is going on?” by GP in G1.1 p5 |
| ***1.2 Something wrong*** | |
| Worsened (‘Rate of change rule’), |  |
| More frequent ( ‘Duration rule’), | Presented because: Repeated episodes  - “The fact I kept on having these episodes” G2.2 p3 by R2 (patient) who talks about repeated aphasia |
| Impacted their day to day activities (‘Severity rule’) | Presented because: Change in impact on life  - R8 G2.2. p12 by GP: “It’s often an impact on life” and they present because partner is worried even if patient themselves are not worried. This makes GP worried as to what has changed. |
| Clearly different from normal (‘Novelty rule’) |  |
| Didn’t understand the changes - uncertainty a trigger to visit a HCP |  |
| Patient agreed to care without hesitation |  |
| *Less common explanations which only sometimes prompted help-seeking* (existing illness, new or existing mental health problems, hormones, virus or infection) | Menopause  - - - G1.2 p10 relative, says wife “was [>50 yrs], that’s the menopause”  Depression  - - - G1.1. p7 “GP said to me that I was depressed” by *patient.*     - R5 G2.2 p21 “Depression can present as cognitive impairment” and GP describes how “people present where they can’t get the words out and just can’t concentrate, it has turned out to be depression”, by *GP.* |
| *Infrequent explanations that prompted help-seeking* (stroke, Alzheimer’s or Dementia, problems with eyesight) | - G1.2 p10, *patient* said “I think you tend to make assumptions perhaps about what is wrong with you” and about pt saying she thought she had Parkinsons.  Alzheimer’s  - - - G1.2 p10, GP “A lot of people … Alzheimer’s, dementia… equally scary for people so they also probably wouldn’t want to go and speak to their GP about that”     - R6 G2.2. p12. Pt says he has family history of Alzheimer’s so he questions whether it’s Alzheimer’s straight away.     - R8 G2.2. p21 “If they’re of an older group, and I’m thinking, is it Alzheimer’s”. by GP  Stroke  - - - Commonly excluded in secondary care       - G1.1. p10     - First differential considered       - “I thought about all the stroke symptoms and went through that, and it just didn’t seem quite that” G2.1 p2 by *relative.*       - “I thought I was having a stroke” R6, G2.1 p6 *patient.*       - Pt thought it was a stroke while having a seizure R2 G2.2 p2 by *patient,* and CT to exclude stroke found the mass.     - G2.2 p12 R7 “When I went to the doctors, they thought it was a stroke” by patient. |
| ***1.3. The social context of symptom appraisal*** | |
| Symptom appraisal occurred within the social context. | - Work colleagues said “I’m getting the same thing, we’re all getting older” and everyone was good at “rationalising” the symptom of not being able to find the words. *By patient.*    - R2 G2.2. p11 also occurred with going into a room and not knowing why they’re there. *Reported by patient.* |
| Not everything was shared |  |
| Family, friends and colleagues encouraged a patient to seek help |  |
| Disagreement - patients did not agree they needed care. |  |
| Decision to seek help was made by others |  |
| **2.0 THE HELP-SEEKING INTERVAL** | |
| ***2.1 Outcome expectations about seeking help*** | |
| Unconvinced that visiting a HCP would be helpful |  |
| Not wanting to waste a GP’s time - did not want to be seen as a hypochondriac, visiting unnecessarily, or too often. ‘Felt silly’ about seeking help, | What stops patients presenting earlier?Fear of wasting resources  - “and they only have 10 minutes and resources are very, very slim on the ground at the moment” by patient relative. G1.1. p10 - R7 G2.2. p10. There is “guilt tripping” in the media with regard to how much appointments cost, etc. Patient: “But in our village magazine we have a regular bit coming from the manager of our healthcare practice. Which gives us a list of how much it cost to refer on. And it costs so much if you come to see us. That is wrong. ” and the response is “it’s guilt tripping”, to which other patients agree. - R4 G2.2. p10, GP. “I as a doctor go to my GP and I feel like I’m so silly.. costing the NHS”  Fear of being a “time-waster”  - G1.2 p10. Pt’s dad was a GP who complained about “all these blooming time-wasters” so pt did not want t be another time-waster - R7 G2.2. p9. Patient: “Oh but I am bothering again”. Response by R1 (relative) on p10 was that most people have this fear and “if those personalities are not strong, the tendency is not to go back” - R4 G2.2. p10. “I as a doctor go to my GP and I feel like I’m so silly.. costing the NHS” - Pt has made a website since the diagnosis where she says “no symptom is small enough” and is encouraging people to present. G2.2 R6. P11. - R9 G2.1 p10 (*either a patient or a relative):* “feeling daft” and fear of doctor thinking that the patient is “stupid” - G2.2 p10. R1, relative: “You don’t want to go and think it’s something trivial, so you don’t want to bother”. - R2 G2.2. p12, patient: “It is … part of being British… let’s do this the right way and chin up and not bother the doctor”.   BUT   - GP (R4 G2.2. p10) says that “I don’t really sit there thinking, this is a bit silly”. - GPs would rather have patient present that to think they are silly. G1.2 p9. Said by GP. |
| ***2.2 Impediments of the Healthcare system*** | |
| Difficulty in accessing prompt care | What stops patients presenting earlier: Lack of resources  - “it’s difficult and they only have 10 minutes and resources are very, very slim on the ground” g1.1. p10, relative - “So, there is, with the funding cuts and everything else, there is only a certain amount of time available and if I go to try and see my GP, there is quite often quite a waiting period.” R1 G2.1 p10 . *RELATIVE..* - R10 G2.1 p12, GP: - talks about having less GPs over the country, short consultation time, lack of resources in general - Difficult to get follow-up appointments   - R1, G2.1 p13, relative: takes 3-5 weeks to get repeat appointment unless doctor specifies that the patient needs double appointments   Presented because: Good relationship with GP  - R2 G2.2 p4 Patient says she has good relationship with GP so would not have hesitated in telling him about her “hangover”-like symptoms. She says that her GP “always had time”. |
| Preference for a specific doctor |  |
| ***2.3 Proximal goals*** | |
| More pressing priorities (e.g. childcare, comorbidities) |  |
